# Retinal Redetachment after Cataract Surgery in Eyes with Previous Scleral Buckling

**Published:** 2011-01

**Authors:** Morteza Mehdizadeh, Mehrdad Afarid, Mohammad Shabanpoor Haghighi

**Affiliations:** Poostchi Ophthalmology Research Center and Department of Ophthalmology Shiraz University of Medical Sciences, Shiraz, Iran

**Dear Editor,**

Removal of the crystalline lens is associated with a substantial increase in the rate of retinal tears and detachments. Retinal detachment (RD) occurs in 2 to 3% of eyes following intracapsular cataract extraction (ICCE), 0.5 to 2 % of eyes following extracapsular cataract extraction (ECCE) but is believed to occur in a smaller percentage of eyes following phacoemulsification. RD frequently occurs within 6 months of cataract surgery or following posterior capsulotomy.^1^,^2^ Predisposing factors for RD after cataract surgery include axial myopia, lattice retinal degeneration, previous RD or retinal tear in the operated eye, history of RD in the fellow eye, and family history of RD.^1^–^3^ An intact posterior capsule appears to be associated with a reduced rate of RD following cataract surgery.

When an ophthalmologist confronts a patient with a history of previous RD requiring cataract surgery, the first question is: how much is the risk of retinal redetachment after cataract surgery?

We designed a prospective interventional case series to evaluate the rate of retinal detachment after cataract surgery in patients with previous rhegmatogenous RD who had been successfully treated by scleral buckling surgery. This study included patients who were referred to the Retina Clinic at Poostchi and Motahari Eye Hospitals between March 2006 and October 2009. These patients had previous rhegmatogenous RD and were treated successfully by various techniques of scleral buckling 1 to 10 years before. Patients who had vitrectomy, pneumatic retinopexy, or multiple operations for RD or techniques other than scleral buckling and those who had developed non-rhegmatogenous RD were excluded from the study. All patients underwent cataract surgery (phacoemulsification or extracapsular surgery with or without an intraocular lens) and were followed for at least one year. The rate of retinal redetachment was evaluated for patients who had completed at least one year of follow up. For patients who had developed posterior capsular opacity during follow up, Nd:YAG laser capsulotomy was performed at least 6 months after cataract surgery. All patients who required laser capsulotomy, were followed for at least one year after the procedure and the rate of retinal redetachment was evaluated.

A total of 21 patients (10 male and 11 female subjects) with mean age of 65.3±7.6 years were included in the study and followed for 1.88±0.49 (range, 1 to 2.5) years. Scleral buckling had been performed from 1 year to more than 10 years prior to cataract surgery at a mean interval of 4.59±3.30 (range, 1 to 10.5) years.

Buckling technique included 360 degree circumferential buckling, segmental buckling, together with the use of sponges or other implants. Phacoemulsification with posterior chamber intraocular lens was performed in 15 patients and extracapsular cataract surgery with an intraocular lens (IOL) was performed for 6 others. For 7 patients, laser capsulotomy was performed due to posterior capsule opacification at least 6 months after cataract surgery. Tears in the posterior capsule or vitreous loss had occurred during cataract surgery in 4 patients. Final BCVA at one year ranged from 20/400 to 20/25. None of the patients experienced RD during the follow-up period. Final visual acuity of 20/40 or better was attained in 5 patients. Of 13 eyes with poor visual acuity (equal to or less than 20/200), 8 had macula-off RD, 4 had glaucoma, and 1 had corneal edema. Risk factors for RD included myopia in 15 patients, myopia with lattice in 5, and trauma in 1 patient.

Our study confirms the results of some previous studies about the safety of cataract surgery in patients who have had previously successful scleral buckling for RD. Although some procedures were complicated by posterior capsule tears and vitreous loss and some eyes required laser capsulotomy, no retinal redetachment occurred.

Haller and Kerrison^4^ reported that eyes which have previously undergone scleral buckling surgery have good visual outcomes after cataract surgery and a low risk of recurrent retinal detachment. The same study showed more intraoperative complications during extracapsular cataract surgery in patients who had previously undergone vitrectomy for RD but a low rate of intraoperative complications in patients with previous scleral buckling.

Smiddy et al^5^ reported no recurrent retinal tears or detachments in patients who underwent extracapsular cataract surgery after previous scleral buckling with an average follow-up period of 24 months.

Ruiz and Saatci^6^ reported a favorable outcome for extracapsular cataract surgery with IOL implantation in eyes that had previously undergone successful scleral buckling. In this study however, 3.4% developed recurrent retinal re-detachment 15 months after cataract surgery.

Eshete et al^7^ demonstrated that phacoemulsification can be performed safely after scleral buckling surgery without any modification in surgical technique. No retinal redetachment was reported in this study.

Shen et al^8^ reported on the safety of phacoemulsification and IOL implantation after scleral buckling surgery and excellent best corrected visual acuity results in most eyes. This study also reported no retinal redetachment.

Tsai and Wu^9^ confirmed the effectiveness of cataract surgery together with scleral buckling, with no significant complications. The authors believed that combined cataract surgery and scleral buckling can improve visualization for detection of peripheral retinal holes and can improve the results of the operation.

According to our study and previous ones, if simple RD is successfully treated with scleral buckling in phakic patients (and all breaks are treated during buckling), when the patients develop cataracts in the future, cataract surgery and laser capsulotomy may be performed without increasing the risk of retinal redetachment.

Eyes with previous RD or predisposing factors for RD are at risk for retinal detachment especially after posterior capsular rupture or after Nd:YAG laser capsulotomy. Mechanisms leading to RD and tears include vitreous traction, traction associated with epiretinal membranes, and fluid movement. Posterior vitreous detachment (PVD) is a predisposing factor for new break formation. Cataract surgery and laser capsulotomy may predispose to PVD and new break formation and subsequently, RD. Scleral buckling surgery decreases the chance of vitreous detachment and prevents the progression of previous PVD by supporting the vitreous base and decreasing the distance between the retina and the vitreous base. Scleral buckling alters the effect of vitreous traction, fluid movement, epiretinal membranes, and also promotes retinal attachment.

This study showed no retinal redetachment in eyes with previously successful scleral buckling. Scleral buckling may protect against break formation or retinal redetachment following cataract surgery or laser capsulotomy, but more cases and long term follow up are required to support this hypothesis. Future studies should compare the rate of retinal redetachment after cataract surgery in eyes with previous vitrectomy versus scleral buckling.
